# P03.03. Facebook use and professionalism among CAM students

**DOI:** 10.1186/1472-6882-12-S1-P256

**Published:** 2012-06-12

**Authors:** G Hinck, R Evans

**Affiliations:** 1 Northwestern Health Sciences University, Bloomington, USA

## Purpose

Social media sites such as Facebook have become a popular way for students to interact, share, and communicate. Studies have found incidents of unprofessional social media use such as posting of protected information or inappropriate photos in both medical and pharmacy students, which has raised concern in healthcare educational settings. To our knowledge, there have been no studies investigating social media use among complementary and alternative medicine (CAM) students.

## Methods

This was an observational study which systematically evaluated the Facebook profiles of all enrolled students at an accredited CAM institution, for type and professionalism of publicly viewable content. Content was deemed unprofessional if there was evidence of alcohol consumption, overt sexuality, foul language or gestures, violence, or patient privacy violations.

## Results

Of 744 students enrolled, identity could be confirmed in 57% with Facebook profiles publicly viewable for 307/492 (chiropractic), 73/116 (oriental medicine) 22/73 (massage) and 23/63 (undergraduate). Unprofessional content was found to a greater degree in undergraduate (48%) and chiropractic (42%) students and less frequently in massage (27%) and oriental medicine (22%). The majority of this unprofessional content involved photos showing alcohol consumption. Patient privacy violations were found in < 1% of sites, only in chiropractic.

## Conclusion

Results indicated that a majority of these CAM students have identifiable Facebook sites and many do not select privacy settings that limit viewing of personal content. Many of these sites contain unprofessional content that could have a negative effect on the reputation and professionalism of the student as well as their program, school, and profession. Posting of unprofessional content varies by program and may be more prevalent in programs with younger students. It is important to understand how our students use social media so that we can develop curricula that support professionalism and responsible use of social media.

